# Congestive heart failure in Indians: How do we improve diagnosis & management?

**Published:** 2010-11

**Authors:** S. Reddy, A. Bahl, K.K. Talwar

**Affiliations:** *Department of Cardiology, Postgraduate Institute of Medical Education & Research, Chandigarh, India*

**Keywords:** Brain natriuretic peptide, device therapy, drug therapy, heart failure

## Abstract

Heart failure is a common cardiovascular disease with high morbidity and mortality. Unlike western countries where heart failure is predominantly a disease of the elderly, in India it affects younger age group. Important risk factors include coronary artery disease, hypertension, diabetes mellitus, valvular heart disease and cardiomyopathies. Plasma brain natriuretic peptide levels are helpful in the diagnosis of heart failure. Echocardiography is the primary imaging modality of choice, through recently cardiac magnetic resonance imaging (MRI) has been found to play an increasing role. Aim of management is to improve symptoms & enhance survival. Diuretics are important in relieving symptoms. Beta-blockers, angiotensin converting enzyme (ACE) inhibitors, angiotensin receptor blockers and adosterone antagonists improve survival in patients with impaired systolic function. Device therapy including cardiac resynchronization therapy and implantable cardiac defibrillators, though expensive are useful in selected patients. Unlike in patients with systolic heart failure where several therapies have been shown to improve survival, clinical trial results in diastolic heart failure have been disappointing and therapy in these patients is restricted to symptom improvement and risk factor control. Therapies like stem cell therapy are being evaluated in clinical trials and appear promising. Early diagnosis and appropriate therapy helps in reversing the process of remodelling and clinical improvement in most of the patients.

## Introduction

Heart failure (HF) is a common cardiovascular condition with increasing incidence and prevalence^1^. Several large clinical trials on use of pharmacological therapy and devices has resulted in an increasing use of evidence based therapy of heart failure. Despite these advances the morbidity and mortality of those afflicted with heart failure continues to remain high. Adherence to guidelines results in improved outcomes of heart failure patients. Education of caregivers on evidence based therapy is the cornerstone of a successful heart failure programme. Unlike western countries where heart failure is predominantly a disease of elderly, in India it affects younger age group. The important risk factors for heart failure include coronary artery disease, hypertension, diabetes mellitus, cardiotoxic drugs, valvular heart disease and obesity^2^,^3^. In India coronary artery disease, diabetes, hypertension, valvular heart diseases and primary muscle diseases are the leading causes for heart failure. Rheumatic heart disease is still a common cause of heart failure in Indians.

## Incidence and Prevalence:

Heart failure is third most common cardiovascular disease in the US affecting 2 per cent of the U.S. population, or almost 5 million people^2^,^3^. The prevalence of heart failure increases with the age from less than 1 per cent in the 20-39 yr old age group to over 20 per cent in the people age 80 yr or older^4^. The life time risk of developing heart failure is estimated at about 20 per cent both in men and women. The lifetime risk of developing HF at the age of 40 yr is 11.4 per cent for men and 15.4 per cent for women. More than 500,000 new cases are diagnosed each year^5^. Around 30 to 40 per cent of patients die from heart failure within 1 year after receiving the diagnosis. Heart failure can be disabling and it can severely reduce a patient’s quality of life.

## Heart failure in India:

In India, we do not have data regarding the exact prevalence and incidence of heart failure. With higher propensity for cardiovascular diseases and ageing population the burden of CHF is likely to be higher in comparison to the western population. Therefore, there is an urgent need to have HF registries in the secondary, tertiary care centers and at the national level. These will help and provide us the detailed information related to heart failure incidence, prevalence, aetiology and thus guide for adopting management strategies.

## Classification of heart failure:

### 

#### Clinical classification :

Heart failure may be either predominantly systolic and diastolic^2^. Patients with systolic heart failure also have some amount of diastolic dysfunction.

#### Diastolic heart failure:

It is estimated that 40 to 50 per cent of patients with heart failure have preserved systolic function or a relatively normal left ventricular ejection fraction. Although such hearts contract normally, relaxation is abnormal. Cardiac output, especially during exercise is limited by the abnormal filling characteristics of the ventricles. These abnormally high filling pressures lead to pulmonary congestion, dyspnoea and oedema^6^–^8^.

Patients with diastolic heart failure are typically elderly, often female, obese and frequently have hypertension and diabetes. Mortality among these patients may be as high as that among patients with systolic heart failure, and the rates of hospitalization in the two groups are similar^9^. The diagnosis of diastolic heart failure is usually made by the typical signs and symptoms of heart failure. Echocardiography aids in the diagnosis of diastolic abnormalities. This entity needs great emphasis and appreciation in clinical practice.

#### Diagnosis of heart failure:

The diagnosis of heart failure is mainly clinical but various investigations help us to understand the underlying cause and assessment of severity of HF. Investigations may help in doubtful cases especially when the clinical presentation is complicated by underlying lung disease.

The classical clinical symptoms of heart failure are exertional dyspnoea, orthopnoea, paroxysmal nocturnal dyspnoea, fatigue and the signs are elevated jugular venous pressure, pulmonary rales, third heart sound and peripheral oedema. No single symptom or sign is pathognomic of heart failure^10^. Clinical findings alone are usually inadequate to differentiate systolic heart failure from diastolic heart failure^10^–^12^.

Symptoms most sensitive for heart failure are exertional dyspnoea, orthopnoea, paroxysmal nocturnal dyspnoea^13^–^16^. The most specific symptoms are orthopnoea and paroxysmal nocturnal dyspnoea. The sensitivity of common symptoms for HF ranges from 23-66 per cent and specificity from 52-81 per cent.

Signs of HF are also non-specific. These include basal crepitations, oedema, raised jugular venous pressure and hepatomegaly. These signs may be absent in patients with compensated heart failure or those on drug therapy. Features of right heart failure are not seen in 50 per cent of the patients with idiopathic dilated cardiomyopathy at initial diagnosis^17^. Valsalva manoeuvre and hepatojugular reflux indicate elevated filling pressures and may be useful in early stages^18^–^20^.

The symptoms and signs bear an inconstant relationship to haemodynamic parameters including resting filling pressures, cardiac index and ventricular function in individual patients. Accurate functional classification remains problematic for individual patients. The NYHA (New York Heart Association) classification is limited by its poor reproducibility and relies exclusively on the presence and severity of symptoms rather any marker of ventricular remodelling.

#### Diagnostic modalities in heart failure:

The ACC/AHA guidelines emphasize *(i)* careful history and physical examination, *(ii)* laboratory investigations including complete blood count, test of renal and hepatic functions, urinanalysis, electrocardiogram and chest x-ray, *(iii)* two dimensional and Doppler echocardiogram *(iv)* careful exclusion of coronary artery disease and thyroid disease in all patients, and (v) selective use of other diagnostic tests including serologic studies in selected patients based upon the clinical characteristics, risk factors, past medical and family history^21^.

#### Electrocardiogram

Electrocardiogram may help to indicate the underlying ventricular hypertrophy or evidence of coronary artery disease. Prolongation of QRS >120 ms occurs in approximately 30 per cent of patients with heart failure^22^,^23^. Left bundle branch block (LBBB) occurs more commonly than right bundle branch block (RBBB) (25 to 36% vs. 4 to 6%, respectively)^24^,^25^. Prevalence of left ventricular (LV) systolic dysfunction increases as QRS complex duration increases progressively above 120 ms^26^.

#### Chest X-ray

Cardiomegaly on skiagram has a sensitivity and specificity of 79 and 80 per cent respectively^27^. Other features on chest X-ray are flow cephalisation, pleural effusion, and pulmonary oedema. Cephalization, interstitial oedema, and alveolar oedema are highly specific (96 to 99%) but insensitive (6 to 41%) markers of acute heart failure^27^.

#### Echocardiogram

Echocardiogram is an important and simple tool to assess the ventricular function and underlying structural abnormality. Assessment of left ventricular systolic function in biplane Simpson’s method is routinely used in clinical practice. It is a valuable tool in measuring LV volumes and assessment of regurgitation. Echocardiography plays a vital role in the diagnosis of patients with heart failure, in part because the physical examination, electrocardiogram, and chest radiograph do not provide information that distinguishes diastolic from systolic heart failure^28^,^29^. Transmitral and pulmonary flow velocities are utilized in the assessment of diastolic dysfunction. Variation in the pattern of these velocities give insight into left ventricular diastolic function and prognosis^30^. Some amount of grade I diastolic dysfunction can be seen in most of the patients and it has to be correlated with the clinical symptoms and signs. Tissue Doppler provides additional information on diastolic dysfunction and myocardial velocities. It differentiates constrictive pericarditis from restrictive cardiomyopathies^31^. Echocardiogram helps in identification of mechanical ventricular dyssynchrony. Echocardiogram should be performed in all patients with symptoms or signs of heart failure as it is a very useful, cheaper, non invasive and easily available in most of the hospitals.

#### Other methods:

Radionuclide cardiac imaging is an excellent modality for assessment of ventricular volumes, geometry, diastolic function and myocardial perfusion. It is commonly used for assessment of myocardial viability. It can differentiate ischaemic and non-ischaemic cardiomyopathy.

CT scan is useful in the evaluation of pericardial disease. Magnetic resonance imaging (MRI) is a validated technique for quantification of volumes, regurgitation and mass of the ventricles. It is a good modality for the assessment of myocardial viability. Myocardial diseases can be diagnosed accurately with cardiac MRI. Experience with cardiac MRI in India is limited since it is not cost-effective for routine evaluation. As coronary artery disease is the leading cause of heart failure, it is preferable to perform coronary angiogram in patients with left ventricular systolic dysfunction who are more than 35 yr of age even in the absence of clinical evidence of coronary artery disease.

#### Role of endomyocardial biopsy (EMB):

0 In dilated cardiomyopathies endomyocardial biopsy is helpful in identification of underlying myocarditis and cardiotropic viruses. In selected cases of infiltrative diseases it can be performed to diagnose the cause when other tests fail to reveal the exact aetiology of heart failure. The current recommendation for its use is in patients with new onset heart failure of < 3 months duration, haemodynamic compromise with or without a dilated ventricle, evidence of arrhythmias and failure to respond to usual care for 1 to 2 wk duration^32^. It is a safe technique. EMB can be obtained through internal jugular or femoral route and 4-5 copies are taken for histopathological, immunochemistry and viral antigen studies.

## Biochemical markers in heart failure

The precursor of brain natriuretic peptide (BNP) and N-terminal pro-brain natriuretic peptide (NT-pro-BNP) is a pre-prohormone BNP, a 134-amino-acid peptide that is synthesized in the myocytes and cleaved to prohormone BNP of 108 amino acids. Prohormone BNP is cleaved by a circulating endoprotease into two polypeptides: the inactive NT-pro-BNP and BNP, a bioactive peptide^33^. BNP and NT-pro-BNP have been shown to distinguish between cardiac and non-cardiac causes of dyspnoea in patients presenting to the emergency and enhances the information provided by the clinical assessment alone^34^–^36^. Two studies comparing NT-pro-BNP and BNP showed that the former was slightly superior to the latter^37^,^38^. The utility of these markers is limited in our routine practice as it is not cost-effective but may be considered in cases of dilemma in differentiation of cardiac or non-cardiac causes of dyspnoea.

## Prevention of heart failure:

### 

#### Primary prevention:

Primary prevention refers to identifying the risk factors for the development of heart failure and their prevention with an aim to reduce the incidence and burden of heart failure. As mentioned earlier the most important risk factors are hypertension, coronary artery diseases, diabetes mellitus and rheumatic heart diseases. Physical activity, dietary control and lifestyle modification can drastically bring down some of these modifiable risk factors and reduce the load of HF.

#### Secondary prevention:

Heart failure can be controlled by both primary and secondary means. Screening through population- based approach and high risk approach can yield satisfactory results and greater reduction in congestive heart failure. Screening for hypertension in individuals more than 30 yr of age and at interval of every 3-5 yr. Screening for diabetic individuals >45 yr of age and persons who have other risk factors for diabetes mellitus. Cholesterol screening (lipid profile) can be performed in individuals >45 yr of age and having risk factors for coronary artery diseases. Screening school children for identification of rheumatic heart diseases, as this is the age group frequently affected. These screening tests can be incorporated into the existing cardiovascular and chronic diseases control programmes. Health education and health promotion strategies are quite important in the success of theses programmes.

#### Community based intervention for prevention of CHF:

There are two types approaches for prevention- high risk approach and population-based approach. High risk approach produces quicker results as seen in a Chinese study in a cohort of 2700 patients (>35 yr of age) with risk factors such as hypertension, diabetes mellitus and coronary artery disease in whom after a period of 3.5 yr the cumulative incidence of stroke was significantly lower in the intervention cohort than the control cohort (0.93 versus 1.34%; RR=0.69; 95% CI, 0.57 to 0.84)^39^. Most major large-scale community-based cardiovascular disease intervention projects use population approach or combination of both high risk and population approach. They all carry out comprehensive interventions activities, involving innovative media campaigns, local media, community participation, co-operation with local and national sectors and policy making. Some of the well planned community intervention projects such as North Karelia Project (Finland), the Stanford Three-Community Study (USA), Stanford Five-City Project (USA), Minnesota Heart Health Program have shown excellent results^40^–^43^. Rather than a single cardiovascular risk factor management a comprehensive cardiovascular risk-management is important as the aetiology of CHF is multifactorial. Cluster of risk factors in a single individual act synergistically increasing the risk more than any one single factor acting alone. The components of intervention in heart failure prevention must include educational approach as well as environmental and policy approach. These community based interventions must target on diet, smoking, physical activity, blood pressure, diabetes mellitus and cholesterol lowering approaches.

## Management of CHF

The management of CHF depends largely on the setting where the patient presents. Almost 75 per cent of the population of India lives in rural areas. Health care in villages are largely provided by the primary health centers from the public health systems and by local primary care physicians who practice myriad form of systems including indigenous system.

**Fig 1:**
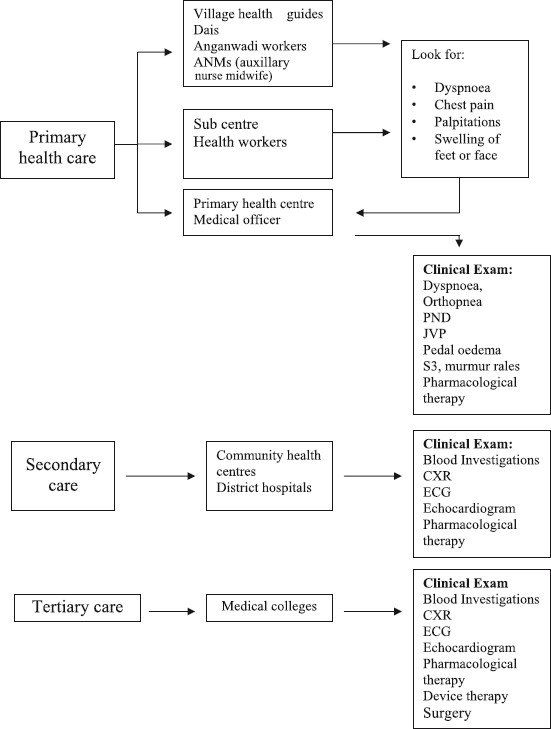
Approach to diagnosis and management of CHF in India health care set up.

In the urban areas again a large proportion of health care is provided by private practitioners and vast expenditure is out of pocket. A few secondary and tertiary centres are available both in the public and private sectors. The management largely depends on where the patient is presenting (Fig. 1).

**Fig 2:**
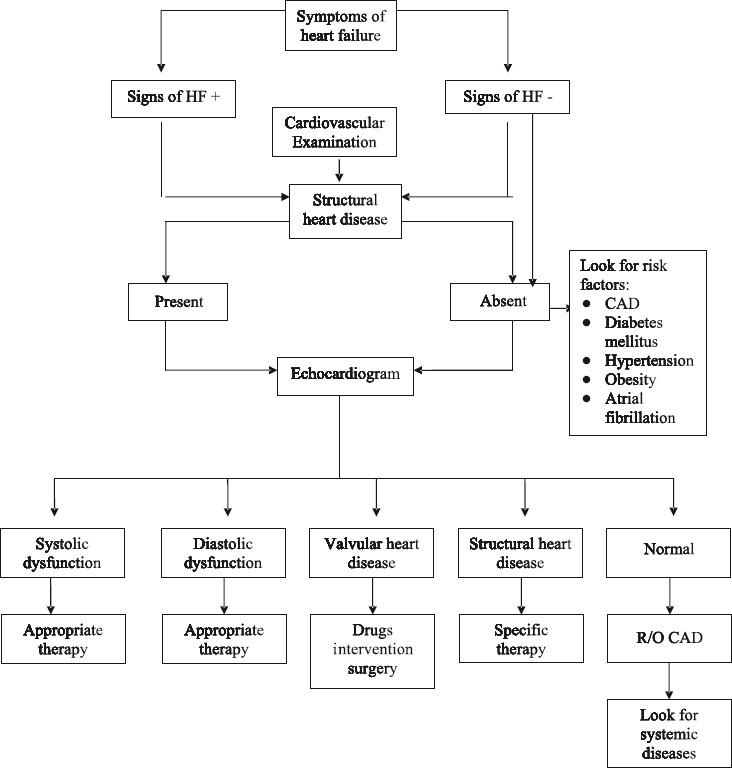
Algorithm for management of heart failure (HF).

The general principles of treatment of heart failure include improvement of symptoms and survival. Every effort has to be made to identify the treatable causes of heart failure and treat the underlying condition. Specific therapy has to be initiated for anaemia, thyroid disorders and nutritional deficiencies. Aggressive management of systemic hypertension, diabetes mellitus, underlying coronary artery disease and surgical correction of valvular heart diseases should be carried out if indicated. Avoidance of alcohol and other toxins is necessary. The detailed algorithm for the diagnosis and management of heart failure is shown in Fig. 2.

Control of heart rate, reversion to normal sinus rhythm and anticoagulation appropriately are the usual measures adopted in patients with atrial fibrillation. In patients with anaemia, characterization of the type of anaemia is important. The role of erythropoietin in patients with normocytic, normochromic anaemia in HF is being evaluated. In bed ridden patients a possibility of pulmonary embolism has to be kept if heart failure worsens in spite of adequate therapy.

Other therapies that are instituted in spite of the specific therapies are similar to all forms of heart failure.

## Non pharmacological treatment of heart failure

*Exercise training:* Exercise training improves exercise capacity and quality of life in patients with mild to moderate heart failure. Early trials have shown beneficial effects of aerobic exercises. More recent trials show that an element of resistance training improves muscle strength, bulk and endurance^44^,^45^. A low level to a moderate intensity (50-80% of maximal capacity) exercise with a warm up period of period of 10 to 15 min, for a duration of 20 to 30 min, 3-5 times a week is recommended in heart failure patients^46^.

*Diet and nutrition:* Heart failure patients are at increased risk of weight loss due to loss of appetite and hypercatabolic status. Adequate calorie intake and nutrients are part of the dietary programmes in these patients. Salt restriction according to symptoms is an essential component. Fluid balance has to be maintained. A supervised nutritional intervention was shown to improve clinical status and quality of life in heart failure patients^47^.

*Education and counselling:* The physician and caregivers should educate the patients regarding the nature of the disease and take steps to prevent further progression of disease. Lifestyle modification and strict compliance to medication is important. Psychological support by the treating physicians boosts the confidence of patients.

## Pharmacological treatment of heart failure

### 

#### Diuretics

These are the main stay of therapy to relieve congestive symptoms. Three classes of drugs are available: loop diuretics, thiazide group and potassium sparing diuretics.

*Loop diuretics*: Agents belonging to this group include furosemide, torsemide and bumetinide. These are most potent diuretics that act on the ascending loop of Henle. Recent data suggest that torsemide and bumetanide are more effective than furosemide in the treatment of advanced heart failure^48^,^49^. Oral absorption of torsemide and bumetanide is complete (ranging from 80-100%) whereas that of furosemide is variable. As furosemide is cheaper compared to other drugs, this is the most commonly used drug in clinical practice. The important adverse effects of these agents on long- term medications are hypokalaemia, hypomagnesemia, hypokalemic alkalosis and orthostatic hypotension.

*Thiazide diuretics*: These act by inhibiting the reabsorption of sodium and chloride from distal convulted tubule. Diuresis with these agents is modest and these are ineffective at glomerular filtration rate below 40 ml/min^48^. The two commonly used agents in clinical practice are chlorothiazide and hydrochlorothiazide. The untoward effects are hypokalaemia, hypomagnesemia, hyperglycaemia, and hyperuricaemia.

*Metalozane* belongs to the quinalozone sulphonamide group. It acts by inhibition of sodium reabsorption in the cortical collecting duct and proximal convoluted tubule. Its main advantage is its efficacy in reduced renal function.

## β-Blockers

### 

The beneficial role of β-blockers in the treatment of heart failure is well established. Agents commonly used in clinical practice are sustained release metoprolol, carvedilol, bisoprolol and nebivolol. Several large randomized placebo-controlled studies in patients with class II-IV heart failure in particular the MERIT-HF, COPERNICUS, CIBIS and COMET trials showed mortality and morbidity benefit with usage of β-blockers^50^–^54^. Non-selective β-blocker, propranalol was found useful in our experience in patients with dilated cardiomyopathy (DCM) with class II-IV symptoms^55^. Current recommendations suggest that the β-blocker therapy should be routinely administered to clinically stable patients with left ventricular systolic dysfunction (LVEF≤40%) and mild, moderate or severe heart failure symptoms^55^. Treatment with β-blockers should be initiated with low doses depending on the clinical status (carvedilol 3.125 mg once or twice daily in severe heart failure). These drugs are to be gradually titrated to maximum doses as tolerated. Clinical response with these drugs may be delayed and it might take 2-3 months to become apparent. The side effects encountered with these drugs are fatigue, bradycardia and hypotension. There is always hesitation in the mind of physicians to start β-blockers in patients with severe heart failure, borderline blood pressure and associated chronic pulmonary diseases. Initiation of β-blockers at a lowest dose possible should be attempted in these group of patients. In those with pulmonary diseases nebivolol may be the preferred agent (starting dose of 1.25 to 2.5 mg once daily).

#### Angiotensin converting enzyme inhibitors (ACEI)

The drugs that produce both venous and arterial dilatation are the preferred agents in heart failure as most forms of heart failure have elevated preload and after load. The ACEI acts on both the arterial and venous capacitance vessels. They act by inhibiting the production of angiotensin II as a consequence preventing the deleterious effects of angiotensin II through its action predominantly on the type 1 receptors. The levels of bradykinin are raised which result in the production of nitric oxide and other important endogenous vasodilators^56^.

There is a clear-cut and compelling evidence that all patients with heart failure and asymptomatic left ventricular dysfunction should receive ACE inhibitor therapy. Multiple well designed prospective randomized placebo-controlled trials, particularly CONSENSUS I, V-HEFT II and SOLVD showed improvement in symptoms and survival in patients with mild to severe heart failure^57^–^59^. The SOLVD trial showed extended benefit of prolonged duration of 12 years^60^. Several trials in heart failure following myocardial infarction including SAVE, AIRE, TRACE and SMILE showed improved survival with ACEI^61^–^64^. These post-infarction trials have demonstrated that ACEI treatment results in a 10-20 per cent reduction in all cause mortality and a 20-50 per cent reduction in the risk of developing symptomatic heart failure. ACEI begun within 24 h of myocardial infarction are safe and are beneficial in whom left ventricular ejection fraction is reduced below 40-45 per cent. The available data do not suggest superiority of any particular ACEI over the other. Thus a cheaper ACEI should be used to enhance compliance. These drugs are initiated at low doses and gradually increased to achieve target doses used in clinical trials. Renal function and serum potassium levels are assessed within 1-2 wk and periodically thereafter. The adverse effects of these agents are dry cough, hypotension, worsening renal function, hyperkalaemia and angioedema.

*Which first- β-blockers or ACEI?:* Historically the efficacy of ACEI in setting of heart failure was demonstrated in the early 1990s, well before the utility of β-blockers was understood. Thus therapy for heart failure is usually started with an ACE inhibitors and β-blockers added later. This practice is not based on any trial evidence but rather appears to be based simply on the historical factors. In fact there is a pathophysiologic basis for the theory that it may be appropriate to start treatment with a beta-blocker. The sympathetic nervous system is activated before the renin-angiotensin-aldosterone system in heart failure, and both systems are inhibited by beta-blockers. Moreover, sudden death, the most prevalent cause of death in early heart failure, is also reduced by β-blockers. The CIBIS III trial compared bisoprolol first and enalapril first regimens. There was no significant difference in the primary endpoint (combined all-cause mortality and all-cause hospitalization) between the two treatment strategies^65^. All cause mortality at the end of the monotherapy phase showed a non significant 28 per cent risk reduction in favour of bisoprolol first at the end of the monotherapy phase and a borderline significant 31 per cent risk reduction in favour of bisoprolol first up to one year. On the other hand, bisoprolol first was associated with 25 per cent increase in risk of worsening heart failure, although this increase was not significant^65^. Results of CIBIS III support a free choice, based on the physician’s individual judgment with each patient, as to whether a beta-blocker or an ACE inhibitor should be used first in patients with heart failure.

#### Angiotensin receptor blockers (ARB)

In the renin angiotensin aldoterone system, the action of the angiotensin converting enzyme produces angiotensin II from angiotensin I. There are other alternative pathways for the generation of angiotensisn II. They are the chymase, chymotrypsin, angiotensin generating enzyme and cathepsin D pathways. The ARBs act at the angiotensisn II receptor level blocking the downstream effects of angiotensin II. ARBs can be used in treatment of heart failure instead of ACE I^66^–^68^. Addition of ARBs to ACE I improves the symptoms and reduce hospitalizations with an added risk of side effects of renal dysfunction and hyperkalaemia^69^–^71^. The combination, however, has not been shown to reduce mortality. Angiotensin converting enzyme inhibitors remains the first choice for the inhibition of the rennin angiotensin system and ARBs are standard alternative in ACE I intolerant patients.

#### Aldosterone antagonists

In heart failure the circulating levels of aldosterone are increased. The deleterious effects include endothelial dysfunction, increased oxidative stress, activation of matrix metalloproteinases, and activation of sympathetic nervous system. In conjunction with angiotensin II enzyme its effects are enhanced. The RALES study showed that spironolactone reduced mortality in severe heart failure patients treated with an ACE I with or without β-blockers by 30 per cent^72^. In the EPHESUS study in patients with heart failure and left ventricular dysfunction following myocardial infarction, there was a significant reduction in mortality and hospitalizations by epleronone^73^. The data from the above studies show that patients with moderately severe or severe heart failure symptoms and recent decompensation or with LV dysfunction following myocardial infarction, addition of aldosterone antagonists may have additional benefits. Spironolactone may cause gynecomastia. In such a situation epleronone may be substituted.

#### Digoxin

Digoxin acts by inhibition of the sodium potassium adenosine triphosphatase enzyme and increases the intracellular Na. This drug is being used for more than a century in the treatment of heart failure. The randomized trials RADIANCE and the DIG trial showed significant reduction in hospitalizations for worsening heart failure but no reduction in mortality^74^,^75^. It is particularly useful in patients of heart failure with atrial fibrillation.

## Therapy in Acute Heart failure

Acute heart failure syndromes (AHFS) are often associated with reversible factors like ischaemia and the cardiac function can return to normal if these are corrected. If not appropriately treated, a vicious cycle is triggered that can lead to chronic heart failure and death. The goals of therapy thus include stabilization of the haemodynamic condition and correction of the underlying reversible factors.

Based on the EFICA and OPTIMIZE HF studies the initiation of treatment in acute heart failure syndromes depend on the systolic blood pressure^76^–^78^. In patients with SBP >140 mmHg the left ventricular function is usually preserved in comparison to patients with SBP <100 mmHg where the systolic function is decreased. Patients with normal or high systolic blood pressure are treated with diuretics and vasodilators such as nitroglycerine and nitroprusside. ACEI or ARB therapy should also be instituted. β-blocker therapy should be started once the patient is haemodynamically stable. Patients with AHFS, low blood pressure and signs of hypoperfusion should receive inotropes.

These patients may need supplemental oxygen therapy to maintain oxygen saturation within normal range by increasing FiO2. In case, oxygen saturation is not maintained, non-invasive positive pressure ventilation (NIPPV) should be considered. NIPPV improves oxygen saturation, decreases the need for endotracheal intubation but does not appear to reduce mortality. Endotracheal intubation and invasive ventilation should be considered if the condition deteriorates.

Some patients may also need temporary mechanical haemodynamic support with intra-aortic balloon pump or left ventricular assist device. Simultaneously, correction of the underlying cause is of primary importance.

## Inotropes in Heart failure

Most inotropic agents act by increasing the myocardial contractility through increase in intracellular cyclic adenylate monophosphate which ultimately leads to increased calcium in the sarcoplamic reticulum. Of these agents, the commonly used in clinical practice are dopamine, dobutamine, ephinephrine, norephinephrine, milrinone and enoximine. Of these inotropes, milrinone and enoximine are phosphodiesterase III inhibitors. Although inotropic agents temporarily stabilize the haemodynamic status, their long-term use is associated with increased mortality^2^,^22^.

## Device Therapy in Heart Failure:

*Cardiac resynchronization therapy:* Cardiac resynchronization therapy (CRT) is an established therapy in patients with LV systolic dysfunction and moderate to severe heart failure symptoms despite optimal medical therapy and evidence of wide QRS > 120 ms^79^. Randomized controlled trials have consistently demonstrated a significant improvement in left ventricular performance, exercise capacity and quality of life. CARE-HF is the first study to show a decrease in all cause mortality with CRT^80^. This has been confirmed in the subsequent two meta-analyses^81^,^82^. The cost of these device is approximately 3.5 lacs and is beyond the reach of many patients. But those who can afford should be referred to centres where these facilities are available.

*Implantable cardiac defibrillator (ICD)*: Sudden cardiac death is responsible for 25 to 50 per cent of mortality in heart failure patients. The percentage of sudden deaths is higher in patients with mild to moderate heart failure as opposed to advanced forms where pump failure accounts for a larger percentage of deaths. The MADIT-II and SCD-HeFT trials confirmed the effectiveness of ICD for primary prevention in heart failure^83^,^84^. In patients with heart failure and LV dysynchrony, the choice of CRT versus CRT-D is individualized as the randomized clinical trial comparing the above therapies did not demonstrate any statistically significant difference between the two treatment arms^85^. The cost of the devices is a limiting factor in the usage of these for primary prevention.

*Ventricular assist devices:* Mechanical circulatory assist devices are used in both acute and chronic heart failure. They are used as a bridge to recovery, bridge to transplant or as permanent destination therapy^86^. Various devices are available which can be used for short term, intermediate or long term. The most radical form of mechanical assist devices is complete orthotopic replacement of native heart with a total artificial heart. Due to cost constraints these device are beyond the reach of patients in a developing country like India.

## Surgery in Heart Failure:

As coronary artery disease is one of the major causes of heart failure every effort has to be made to undertake complete revascularization after assessment of myocardial viability. In patient with valvular heart diseases repair or replacement as per recommendations and guidelines is helpful in prevention and treatment of heart failure. In patients with secondary or functional mitral regurgitation and ischaemic mitral regurgitation, valve repair surgery preserving the mitral apparatus is advocated. Other surgical modalities reshape the dilated ventricle to decrease the volume and stress on the ventricle. Partial left ventriculectomy (Batista procedure) is no longer popular due to poor long-term outcomes. Cardiac transplantation remains the gold standard therapy for end stage heart disease^87^,^88^.

## Cell therapy in heart failure:

Studies have demonstrated modest increase in LV ejection fraction and improvement in symptoms in patients with LV dysfunction of both ischaemic and non ischaemic aetiology^89^–^91^. Several studies using cell therapy of various cell lineage in patients with LV dysfunction following myocardial infarction showed encouraging results^92^–^95^. At present, this is not an established therapy in heart failure patients. Currently a randomized clinical trial is under way and is conducted by the DBT (Department of Biotechnology), Government of India, involving six centres in India (CMC, Vellore; PGIMER, Chandigarh; SGPGI, Lucknow; Army Hospital, New Delhi; CTC, Pune and AIIMS, New Delhi) to study the effect of bone marrow mononuclear cells in patients with acute anterior wall myocardial infarction and LV dysfunction.

## Heart Failure Programmes:

Disease management programmes (DMP) have been developed to standardize and optimize CHF treatment. These programmes focus on education of the patient and continuing support after hospital discharge. Studies have shown that these programmes have been shown to reduce mortality and hospitalization rates, improve quality of life and economically beneficial.

## Telemonotoring in Heart failure:

Telemedicine is being increasely used in India in the diagnosis and management of disease to people out of reach in remote areas. Telemonitoring can also be effectively used in monitoring heart failure patients. By allowing clinical data to be collected without the need for face to face contact with the patients, telemonitoring facilitates easy access for management and therby improving the clinical outcome.

There are several ways by which telemonitoring can be done. These are telephone based symptom monitoring, automated physiologic monitoring and automated monitoring of symptoms and signs. Trained nurses can collect the required information which can later be reviewed by physicians. In the automated systems the information regarding the symptoms, signs and physiological parameters are uploaded into the particular websites which are centrally located and monitored by heart failure specialists, cardiologists and or physicians.

## Conclusions

Heart failure in India has reached epidemic proportions. Early identification of the risk factors and initiation of appropriate therapy at early stages prevents development of heart failure. Clinical diagnosis and diagnostic imaging, echocardiogram in particular identifies patients with heart failure. The optimum utilization of the available drugs, general measures and surgical procedures appropriate to the condition improves the outcome of these patients.
